# Metabolic potential and contributions of ammonia-oxidizing microorganisms and complete ammonia oxidizers to soil nitrification in upland soils of northern China

**DOI:** 10.1093/ismeco/ycag122

**Published:** 2026-05-07

**Authors:** Jiuwei Song, Qichun Zhang, Ahmed A A Aioub, Longda Gong

**Affiliations:** State Key Laboratory of Soil Pollution Control and Safety, Zhejiang Provincial Key Laboratory of Agricultural Resources and Environment, Zhejiang University, Hangzhou 310058, Zhejiang Province, PR China; Jiangsu Key Laboratory for Bioresources of Saline Soils, Jiangsu Provincial Key Laboratory of Coastal Wetland Bioresources and Environmental Protection, Jiangsu Synthetic Innovation Center for Coastal Bio-agriculture, Yancheng Teachers University, Kaifang Road 50#, Yancheng 224002, Jiangsu Province, PR China; State Key Laboratory of Soil Pollution Control and Safety, Zhejiang Provincial Key Laboratory of Agricultural Resources and Environment, Zhejiang University, Hangzhou 310058, Zhejiang Province, PR China; Plant Protection Department, Faculty of Agriculture, Zagazig University, Zagazig 44511, Sharqia Governorate, Egypt; State Key Laboratory of Soil Pollution Control and Safety, Zhejiang Provincial Key Laboratory of Agricultural Resources and Environment, Zhejiang University, Hangzhou 310058, Zhejiang Province, PR China

**Keywords:** ammonia-oxidizing bacteria, ammonia-oxidizing archaea, comammox, soil nitrification

## Abstract

Ammonia-oxidizing bacteria (AOB), ammonia-oxidizing archaea (AOA), and complete ammonia oxidizers (comammox) play a crucial role in nitrification, which is an essential process in terrestrial nitrogen cycling. However, their metabolic potential and relative contributions to soil nitrification in upland soils remain unclear. In this study, the metabolic pathways and the interaction of AOA, AOB, and comammox of upland soil in northern China was conducted by combination of ^13^C-stable isotopes and multiple inhibitor methods. Meanwhile, under acetylene inhibition, the urea treatment in WI2 soil exhibited remarkably high ^13^C labeling proportions in AOA and comammox, reaching 80.3% and 64.2%, respectively, compared with the other soils. This suggests that long-term application of organic liquid fertilizer may induce shifts in microbial community composition and functional potential. Furthermore, inhibition of AOA reduced the ^13^C-labeled abundance of comammox, indicating that comammox may depend on metabolites produced by AOA. Across all soil treatments (CK, WI1, and WI2), the nitrification potential of AOA and AOB acting together was lower than the sum of their respective nitrification potentials when operating independently. However, in the WI2 treatment, the co-occurrence of AOA and AOB increased the nitrification potential of comammox from 22%–26% to 29%–46%, indicating that the presence of AOA and AOB can enhance the nitrification potential of comammox in upland soils with high organic matter content. The results of this study provide the knowledge of the genetic and metabolic associations among soil nitrifying microbial communities.

## Graphical Abstract

**Graphical Abstract:**
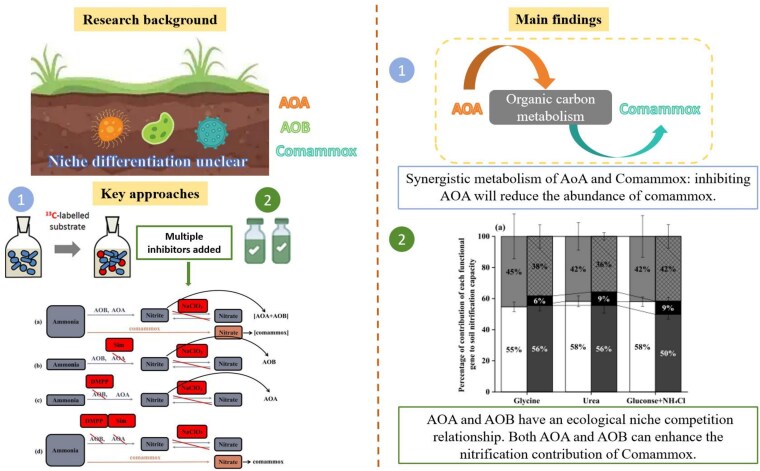

##  Introduction

Nitrification is a key oxidation process in the nitrogen cycle [1, 2]. Its initial step, ammonia oxidation, has traditionally been attributed to the combined activity of ammonia-oxidizing bacteria (AOB) and ammonia-oxidizing archaea (AOA) [3]. However, the discovery of complete ammonia oxidizers (comammox) in 2015 fundamentally redefined this paradigm, as these organisms are capable of fully oxidizing ammonia to nitrate independently [4]. All comammox strains identified to date belong to clade II of the genus *Nitrospira*, and they are widely distributed and abundant in soils, with a competitive advantage particularly in oligotrophic and slow-growing environments [5]. Notably, comammox exhibits exceptional metabolic versatility: it can hydrolyze cyanate and urea [6], and utilize formate or hydrogen as alternative energy sources via oxidation [7, 8]. Nevertheless, the potential for organic carbon metabolism in comammox, as well as the quantitative assessment of its contribution to nitrification, remains largely unexplored [9].

In contrast to the obligate autotrophy of AOB, AOA exhibit greater metabolic flexibility in their carbon assimilation and energy utilization strategies. Evidence suggests that AOA may enhance ammonia oxidation activity through intermediates of the tricarboxylic acid (TCA) cycle, such as α-ketoglutarate [10], and potentially utilize organic carbon even under CO₂-limiting conditions [11]. Genomic analysis further supports the heterotrophic potential of AOA, which has the ability to convert carbohydrates [12], and some strains are capable of integrating carbon metabolism through both the 3-hydroxypropionate/4-hydroxybutyrate cycle and the TCA cycle [13]. Nonetheless, there is ongoing debate on AOA’s ability to metabolize complex organic substrates, including the functional completeness of the TCA cycle, and its metabolic coupling with comammox [14, 15].

The functional linkages and competitive relationships among ammonia-oxidizing microbes are further highlighted by the discovery of comammox. The functional differentiation between AOA and AOB has been widely studied: AOB prefers neutral to alkaline high-ammonia environments, while AOA has more advantages in acidic low-ammonia soils [16, 17]. Comammox’s specialty traits, on the other hand, are still unknown. Previous research suggests that ammonium nitrogen content [18], phosphorus availability [19], and oxygen demand [20] may all work together to control its dispersion. It is yet to be determined how the three different kinds of microbes interact and whether their contributions to nitrification are competitive or synergistic in the process of organic matter metabolism.

To elucidate the relative functional contributions of different ammonia-oxidizing microbial groups in soil nitrification, previous studies have commonly combined multiple chemical inhibitors to operationally regulate specific steps or functional guilds within the nitrification process. It should be noted that in complex soil systems, the selectivity of chemical inhibitors cannot be considered absolute, as their inhibitory effects may vary depending on soil properties and experimental conditions [21]. Therefore, in this study, inhibitors were used as experimental tools to compare response patterns among treatments rather than to completely distinguish specific microbial groups.

Acetylene (Ace) is widely used to inhibit autotrophic nitrification and is considered to substantially suppress ammonia-oxidizing activity [22, 23]. Under certain conditions, it may affect both AOB and AOA [24]. Simvastatin (Sim) has been employed in previous studies to preferentially regulate AOA-related ammonia oxidation, with relatively weaker direct effects on AOB and comammox [17, 25]. The 3,4-Dimethylpyrazole phosphate (DMPP) is commonly used to inhibit AOB-associated nitrification, although its inhibitory efficiency may vary with soil type [26]. In addition, sodium chlorate (NaClO_3_) primarily inhibits the second step of nitrification, namely the oxidation of nitrite to nitrate, while exerting limited influence on the initial ammonia oxidation step [27]. Based on these considerations, Ace, Sim, DMPP, and NaClO_3_ were jointly applied in this study as operational tools to compare the response characteristics of nitrifying microbial communities under different soil treatments.

This study integrates DNA-based Stable Isotope Probing (DNA-SIP) with a refined multi-inhibitor strategy to address two fundamental questions:

It remains unclear whether AOA and comammox exhibit metabolic interdependence during organic carbon utilization;

How AOA, AOB and comammox partition their contributions to soil nitrification and interactively regulate nitrification processes.

## Materials and methods

### Study sites and soil sampling

Soil samples were collected from upland soil in Ningxia, China (35°49′54″ N, 106°40′29″ E) (Supplementary Fig. S1). The sites has a temperate continental climate with mean annual temperature and precipitation of 8.5°C and 450 mm, respectively. The upland soil classification is a coarse-textured sierozem. The sites has three treatments including CK (control; no fertilizer), WI1 (an annual application of 80 m^3^ ha^−1^ liquid organic fertilizer delivered to the soil through drip irrigation in ten equal doses for 1 year), and WI2 (an annual application of 80 m^3^ ha^−1^ liquid organic fertilizer delivered to the soil through drip irrigation in ten equal doses for 2 years). The total nitrogen content of the organic liquid fertilizer used in this study was 4.2 g·l^−1^, including 3.8 g·l^−1^ organic nitrogen (mainly in the form of amino acid–N) and 0.4 g·l^−1^ NH_4_^+^–N. Soil samples were collected from the 0–20 cm surface layer using a random sampling method. The samples were then passed through a 2.0-mm sieve to remove stones, animals, large root fragments, and plant materials. One portion of each sample was stored at −80°C until DNA extraction, while the other was air-dried at ~25°C until soil physicochemical property analyses and incubation.

### Isotope tracer incubation

To investigate the metabolic roles of AOA and comammox, ^13^C-glycine (^13^C-Gly), ^13^C-urea (^13^C-Urea), and ^13^C-glucose plus ammonium chloride (^13^C-Glu + Amm) were used as carbon and nitrogen sources in three soil treatments (CK, WI1, and WI2)，and the Ace and Sim were applied to inhibit autotrophic nitrification and AOA, respectively. All chemicals used in this study were of analytical grade unless otherwise stated. Ace, Sim, DMPP, and NaClO_3_ were obtained from Sangon Biotech (Shanghai) Co.,Ltd. ^13^C-Urea, ^13^C-Gly, and ^13^C-Glu + Amm (99 atom% ^13^C) were purchased from Beijing Innochem Science & Technology Co.,Ltd. (China). The following treatments were conducted: (i) ^13^C-Gly, (ii) ^13^C-Gly + Ace, (iii) ^13^C-Gly + Sim, (iv) ^13^C-Urea, (v) ^13^C-Urea+Ace, (vi) ^13^C-Urea+Sim, (vii) ^13^C-Glu + Amm, (viii) ^13^C-Glu + Amm + Ace, and (ix) ^13^C-Glu + Amm + Sim. The rate of ^13^C-Gly, ^13^C-Urea and ^13^C-Glu + Amm addition were 100 mg N kg^−1^, 100 mg N kg^−1^, 200 mg C kg^−1^ + 100 mg N kg^−1^, respectively (Supplementary Table S1). The Ace and Sim were applied at rates of 1% (v/v), 12.5 mg g^−1^, respectively. Every ^13^C treatment has a matching ^12^C control treatment to show the marking situation of ^13^C. Then, 20 g fresh soil was transferred to each 120 ml serum vial, and the soil water content was adjusted to 50% WFC. The vials were incubated at 25°C for 4 weeks, and destructive soil sampling was conducted at 0, 3, 7, 15, and 28 days of incubation.

### Determination of soil nitrification potential

**Figure 1:**
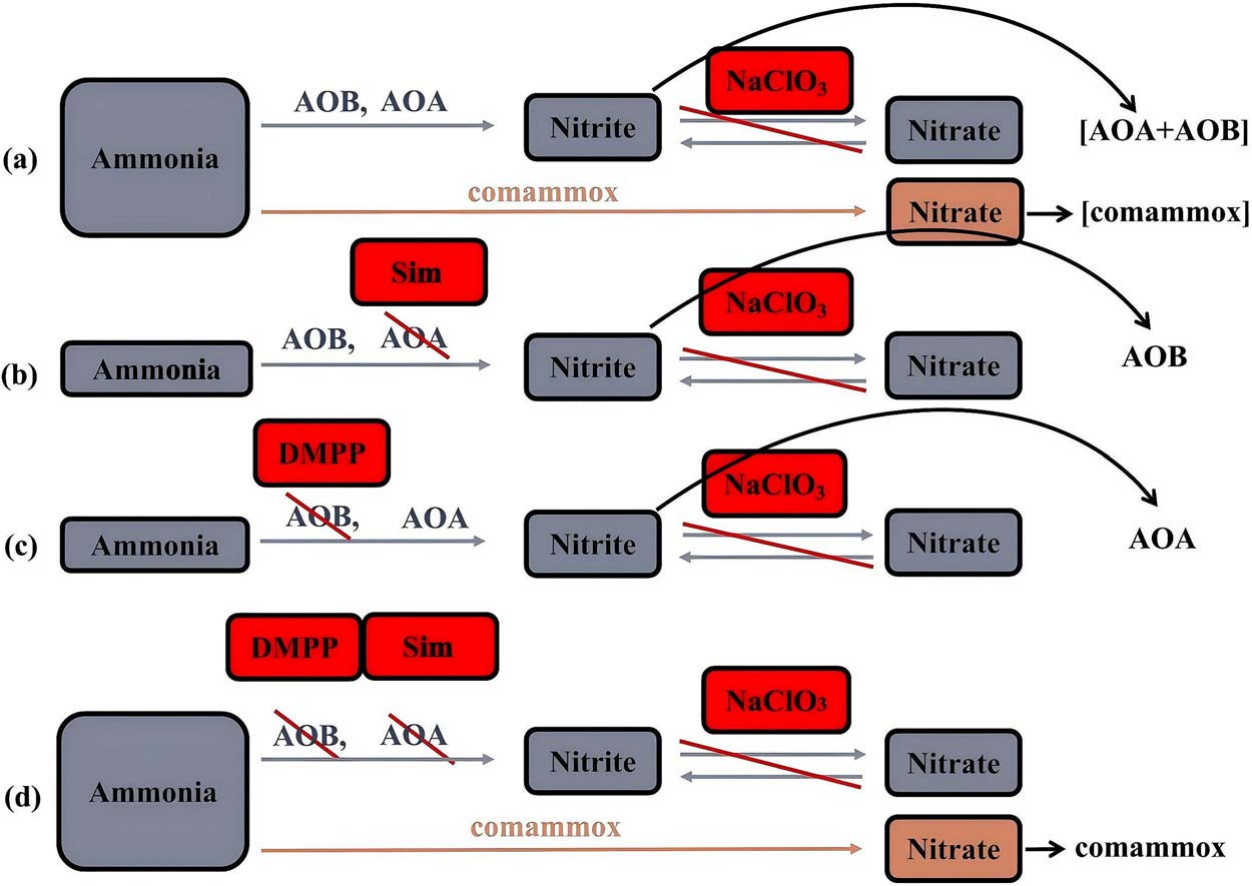
Schematic diagram of determination of single nitrification potential of AOA, AOB or comammox, respectively. Sim: simvastatin; DMPP: 3,4-Dimethylpyrazole phosphate. On the right side, AOA, AOB, and comammox represent their respective individual contributions to nitrification potential, and the [AOA + AOB] reflects the combined contribution of AOA and AOB; the [comammox] represents the contribution of comammox when AOA and AOB are not inhibited.

The relative contributions of AOA, AOB, and comammox to soil nitrification were distinguished using a multiple-inhibitor approach (workflow illustrated in Fig. 1). Under these conditions, the accumulation of NO_2_^−^ reflects the combined contribution of AOA and AOB (denoted as [AOA + AOB], Fig. 1a), while the increase in NO_3_^−^ concentration represents the contribution of comammox when AOA and AOB are not inhibited (denoted as [comammox], Fig. 1a). The inhibition of AOA and AOB was achieved by the addition of Sim and DMPP, respectively. Thus, the contributions of AOA or AOB were determined by measuring NO_2_^−^ accumulation following the addition of NaClO_3_ plus Sim (denoted as AOB, Fig. 1b) or NaClO_3_ plus DMPP (denoted as AOA, Fig. 1c). The contribution of comammox under full inhibition of AOA and AOB was assessed by measuring NO_3_^−^ concentrations following the addition of all three inhibitors (denoted as comammox, Fig. 1d). The application rates of the different inhibitors used in the soil nitrification potential assay are provided in Supplementary Table S3.

Nitrification potential was assessed using ^13^C-labeled soils after incubation (section 2.2). Soils without inhibitor treatment (Ace or Sim) were used as the experimental group, while Ace-treated soils served as controls to account for background heterotrophic nitrification. Soil nitrification potential was determined via a shaken soil-slurry method [28], allowing quantification of the relative contributions of AOA, AOB, and comammox. Specifically, 1 mM phosphate buffer was amended with: (i) 10 mM NaClO_3_, (ii) 10 mM NaClO_3_ + 12.5 g L^−1^ Sim, (iii) 10 mM NaClO_3_ + 1.5% (w/v) DMPP, or (iv) 10 mM NaClO_3_ + 12.5 g L^−1^ Sim + 1.5% (w/v) DMPP. Then, 10 g of CK, WI1, or WI2 soil was added to 100 ml phosphate buffer (containing 1.5 mM NH_4_^+^ and 1 mM PO₄^3−^) in a 250 ml conical flask fitted with a ventilated lid. Flasks were shaken at 180 rpm for 24 h. During incubation, 10 ml slurry samples were collected at 2, 4, 8, 20, 22, and 24 h, centrifuged at 4200 rpm for 5 min at 25°C, and the supernatant filtered through a 0.45-μm membrane. Nitrate (NO_3_^−^) and nitrite (NO_2_^−^) concentrations were immediately measured. Nitrification potentials (expressed as NO_3_^−^-N d^−1^ or NO_2_^−^-N d^−1^) were calculated based on the linear increase of NO_3_^−^ and NO_2_^−^ concentrations over time. Concentrations of NO_3_^−^ and NO_2_^−^ were determined spectrophotometrically following the methods described by Pappenhagen [29] and Barnes [30], respectively. A schematic diagram showing the determination of the individual nitrification potentials of AOA, AOB, and comammox is shown in Fig. 1. The nitrification potential was calculated according to Eq. 1:


1 \begin{eqnarray*}Np=R\times\frac{0.1+V}{m}\times 24,\end{eqnarray*}

where *Np* is the soil nitrification potential (mg kg^−1^ d^−1^), *R* is the rate of increase in NO_2_^—^N or NO_3_^—^N concentration (mg l^−1^ h^−1^), 0.1 is the buffer volume (l), *V* is the water volume in the soil sample (l), and *m* is the soil dry mass (kg).

### Soil physicochemical analysis

The physicochemical characteristics of soil are displayed in Table 1. The pH values of 1:2.5 (v/v) soil: CaCl_2_ suspensions were measured using a pH meter (Mettler-S210 SevenCompact™). Soil available phosphorus (Olsen-P) was determined by the Mo-Sb colorimetric method using soil extracted with 0.5 M sodium bicarbonate (NaHCO_3_). Soil available potassium was determined by flame photometry (FP640; INASA, Shanghai, China) using soil extracted with 1 M ammonium acetate (NH_4_OAC). Soil total C and total N in dry soil soaked in 0.1 M hydrochloric acid (HCl) were determined with an elemental analyzer (Vario MAX; Elementar Analysensysteme GmbH, Langenselbold, Germany). Soil NO_3_^−^, NH_4_^+^, and NO_2_^−^ were extracted with 2 M potassium chloride (KCl) and determined using the Griess reagent, indophenol, and *N*-1-(naphthyl)-amine light methods, respectively [31].

**Table 1:** Soil physicochemical properties.

Soil	**Olsen-P** mg kg^−1^	**Av-K** mg kg^−1^	**OM** g kg^−1^	pH	**NH**_**4**_ ^**+**^ mg kg^−1^	**NO**_**3**_ ^**−**^ mg kg^−1^	**TC** %	**TN** %
CK	36.5 ± 2.3c	95.3 ± 2.5a	3.8 ± 0.05b	7.7 ± 0.2b	0.6 ± 0.1b	1.8 ± 0.2b	0.22 ± 0.05b	0.045 ± 0.002a
WI1	40.6 ± 1.2b	94.2 ± 1.3a	4.1 ± 0.03b	7.7 ± 0.1b	0.8 ± 0.2a	5.2 ± 0.6a	0.24 ± 0.03b	0.042 ± 0.003a
WI2	42.8 ± .4a	96.5 ± 5.2a	5.0 ± 0.04a	7.9 ± 0.1a	0.8 ± 0.2a	4.8 ± 0.3a	0.29 ± 0.04a	0.047 ± 0.002a

Olsen-P: available phosphorus; Av-K: soil available potassium; OM: (soil) organic matter; TM: total nitrogen; TC, total carbon. Different letters indicate significant differences among soil types (*P* < .05). Error bars indicate standard deviation (SD).

### DNA extraction and *amo*A quantification

Soil DNA was extracted with an E.Z.N.A.® Soil DNA Kit (Omega Bio-tek, Norcross, GA, USA) according to the manufacturer’s instructions. DNA quantity and quality were evaluated using a NanoDrop spectrophotometer (Thermo Fisher Scientific, Waltham, MA, USA). Archaeal, bacterial, and comammox *amo*A abundances were estimated by quantitative polymerase chain reaction (qPCR) in a StepOnePlus Real-Time PCR System (Applied Biosystems, Foster City, CA, USA). The primers used for *amo*A detection are listed in Table 2. The PCR reactions were performed in 20 μL reaction volume comprising 10 μL QuantiTect SYBR® Green Master Mix (Qiagen, Hilden, Germany), 0.5 μL of each 10-μM forward and reverse primer, and 1 μL DNA template (5 ng μL^−1^). To verify nonspecific amplification, a melting curve analysis was conducted by increasing the temperature from 60°C to 95°C at an increment of 4.4°C s^−1^. The numbers of gene copies were calculated according to a standard curve plotted using standard plasmid DNA.

**Table 2:** Primers and conditions for each functional gene.

Target gene	Primer	Primer sequence (5'-3')	Amplification conditions
AOA	Arch-amoaAF	STAATGGTCTGGCTTAGACG	95°C for 5 min; 94°C for 20 s, 55°C for 1 min, 72°C for 30 s; 35 cycles
	Arch-amoaAR	GCGGCCATCCATCTGTATGT	
AOB	AomA-1F	GGCGTTTCTACTGGTGGT	95°C for 5 min; 94°C for 30 s, 55°C for 30 s, 72°C for 1 min; 35 cycles
	AmoA-2R	CCCCTCKGSAAAGCCTTCTTC	
Comammox	Ntsp-amoA 162F	GGATTTCTGGNTSGATTGGA	95°C for 5 min; 94°C for 30 s, 48°C for 30 s, 72°C for 1 min; 40 cycles
	Ntsp-amoA 359R	WAGTTNGACCACCASTACCA	
NOB	nxrB169f	TACATGTGGTGGAACA	95°C for 5 min; 94°C for 30 s, 56°C for 40 s, 72°C for 90 s; 35 cycles
	nxrB638r	CGGTTCTGGTCRATCA	

AOA: ammonia-oxidizing archaea； AOB: ammonia-oxidizing bacteria； NOB: nitrite-oxidizing bacteria

### Stable isotope probing, functional gene sequencing, and phylogenetic analysis

DNA was extracted from microcosm samples after 28 days of incubation and subjected to isopycnic density gradient centrifugation. DNA-SIP was conducted following the detailed protocol described by Neufeld *et al.* [32]. Briefly, 1 μg DNA was mixed with 1.710 g ml^−1^ CsCl in Tris-EDTA buffer in an 8 ml quick-seal polyallomer tube (Beckman Coulter, Brea, CA, USA) and then centrifuged in a Vti65.2 vertical rotor (Himac CP80NX; Hitachi Koki Co. Ltd., Tokyo, Japan) at 100 000 × *g* (45 000 rpm) and 20°C for 44 h. The DNA was then separated into 15 500-μL fractions. The DNA in each fraction was precipitated by incubation with 1 ml polyethylene glycol (PEG-6000) and 10 μg glycogen (Thermo Fisher Scientific) at 4°C overnight, followed by centrifugation at 16 000 × *g* and 4°C for 45 min. Each DNA pellet was washed with 1 ml of 70% (v/v) ethanol and resuspended in 30 μL sterile water. The archaeal, bacterial, and comammox *amo*A in DNA fractions 2–14 were quantified by qPCR. Ammonia oxidizer activity was determined by comparing the ^12^C-DNA and ^13^C-DNA incorporation profiles when the relative gene abundance was higher in the ^13^C-DNA- than the ^12^C-DNA-amended treatments in the heavy fractions. The AOA, AOB, and comammox activity levels were calculated as the proportions of cells incorporating ^13^C-DNA multiplied by the total corresponding *amo*A abundance in each sample estimated by qPCR. The heavy fraction DNA was sequenced for the AOA and comammox *amo*A; primers are listed in Table 2. Raw sequencing data were clustered at 100% best identity by QIIME2 (qiime.org) to produce an amplicon sequence variant (ASV) feature table. The ASV were checked for chimeras and aligned with reference sequences using MUSCLE in MEGA7 (https://www.megasoftware.net) [33]. Phylogenetic analysis was performed by constructing a neighbor-joining tree using the translated amino acid sequences and the Kimura 2-parameter distance with 1000 replicates to produce bootstrap values. The nucleotide sequences were deposited at the National Center for Biotechnology Information database (https://www.ncbi.nlm.nih.gov/searc) under Registration No. PRJNA819570.

### Data analysis

One-way ANOVA and Duncan’s test were executed using SPSS v. 20 (SPSS Inc., Chicago, IL, USA) to compare functional gene abundances, NH_4_^+^ and NO_3_^−^ concentrations, and percentage of nitrification among treatments. The relative proportions and abundances of the ^13^C-labeled AOA, AOB, and comammox genes in the heavy fractions between corresponding microcosms were analyzed in SPSS v. 20 using independent Student’s *t*-tests. Differential abundance analysis of ASVs was conducted using the Wald test implemented in DESeq2, with a significance threshold of *P* < .05. All other statistical analyses were performed and results were plotted using R v. 3.6.1 (R Core Team, 2019) and Origin 2020 (OriginLab, Northampton, MA, USA), respectively. All data are presented as the mean values of three biological replicates.

## Results

### Changes in soil NH_4_^+^, NO_3_^−^ concentrations and nitrifier abundance during incubation

**Figure 2:**
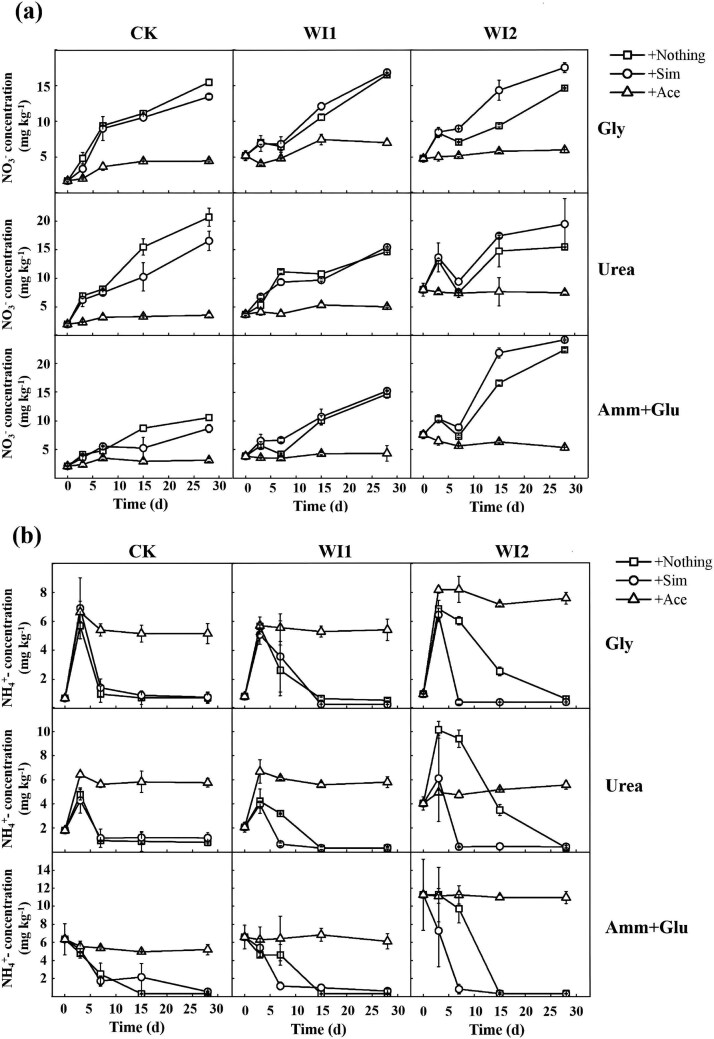
Changes in NO_3_^−^ (a) and NH_4_^+^ (b) concentration in each treatment during incubation. Sim: simvastatin; Ace: acetylene; Gly: glycine; Glu+Amm: glucose plus ammonium chloride. Error bars indicate standard deviation, *n* = 3.

To evaluate the effects of different carbon sources and inhibitors on nitrification, we compared changes in NH_4_^+^ and NO_3_^−^ across treatments. Under Ace inhibition of nitrification, NH_4_^+^ responded differently depending on the substrate. NH_4_^+^ concentrations increased in the ^13^C-Gly + Ace and ^13^C-Urea+Ace treatments, indicating ammonium accumulation when nitrification was suppressed, whereas no clear change was observed in the ^13^C-Glu + Amm + Ace treatment (Fig. 2b). In the absence of Ace, NO_3_^−^ increased across treatments, suggesting active nitrification. A slight increase was observed in CK soil, little change in WI1, and a more pronounced increase in WI2 following Sim addition, indicating that differences in soil background and inhibitor treatments may alter the relative contributions of nitrifying communities.

**Figure 3:**
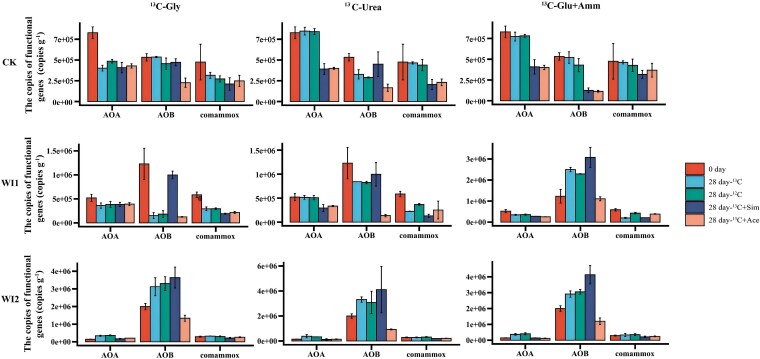
Changes in the abundance of AOA, AOB, and comammox for each treatment at the beginning and end of the incubation. Sim: simvastatin; Ace: acetylene; Gly: glycine; Glu+Amm: glucose plus ammonium chloride. Different letters indicate significant differences between treatments at *P* < .05, *n* = 3.

The abundances of AOA, AOB, and comammox were further quantified by qPCR across different treatments (Fig. 3). In CK soil amended with ^13^C-Urea or ^13^C-Glu + Amm, AOA abundance was relatively high but declined after inhibitor addition. AOB abundance followed the pattern WI2 > WI1 > CK and was dominant in WI2 soil; Sim increased AOB abundance, whereas Ace decreased it. The abundance pattern of comammox was similar to that of AOA, with the highest levels in CK and the lowest in WI2. These results indicate that different carbon inputs and inhibitor treatments did not produce a uniform response pattern; rather, they reshaped nitrification processes across soil types by altering nitrogen accumulation patterns and the relative abundance of different ammonia-oxidizing groups.

### Substrate utilization traits of nitrifying microorganisms

**Figure 4:**
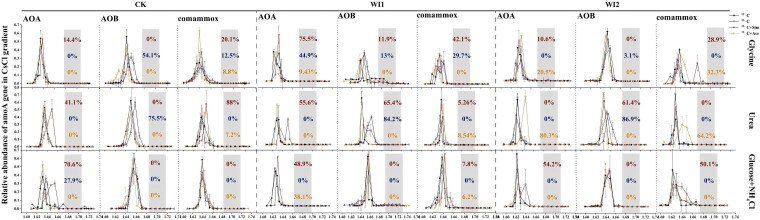
Relative abundances of archaeal, bacterial, and comammox amoA genes in CsCl gradients of ^13^C and ^12^C-DNA. The gray area represents the operationally defined heavy DNA fraction along the CsCl density gradient, where ^13^C-labeled DNA was enriched relative to the ^12^C control. Red, blue, and yellow highlights within this region indicate the relative abundances of ^13^C-labeled archaeal, bacterial, and comammox amoA genes, respectively, which were used for quantifying ^13^C incorporation. Sim: simvastatin; Ace: acetylene; Gly: glycine; Glu+Amm: glucose plus ammonium chloride. Vertical error bars represent standard errors of means of triplicate relative abundances.

The incorporation characteristics of nitrifying microorganisms for different organic carbon sources were explored by using DNA-SIP technology (Fig. 4). In CK soil, the abundance of ^13^C-labeled AOB did not show a clear response to the addition of different ^13^C-labeled organic substrates, indicating that AOB made a limited contribution to carbon assimilation under the unfertilized background. When Sim was added to CK to suppress AOA, the proportion of ^13^C labeling declined markedly and varied among substrates: labeling in response to Gly and urea was completely abolished, while the response to NH_4_Cl was substantially reduced (Supplementary Table S2). These results indicate that in CK soil, AOA and comammox were the primary contributors to isotope incorporation, and their activity was strongly inhibited by Sim. In long-term fertilized soils (WI1 and WI2), except for the glucose treatment, the abundances of ^13^C-labeled AOA, AOB, and comammox were generally higher than in CK (Fig. 4), suggesting that fertilization enhanced the responsiveness of ammonia-oxidizing groups to external substrates. After Ace was added to inhibit nitrification, ^13^C labeling of AOA and comammox in WI1 was lower than in WI2; however, in WI2 soil, 80.3% and 64.2% of AOA and comammox, respectively, were still ^13^C-labeled. This indicates that under long-term organic input, portions of these populations maintained relatively high carbon assimilation even when nitrification was suppressed.

**Figure 5:**
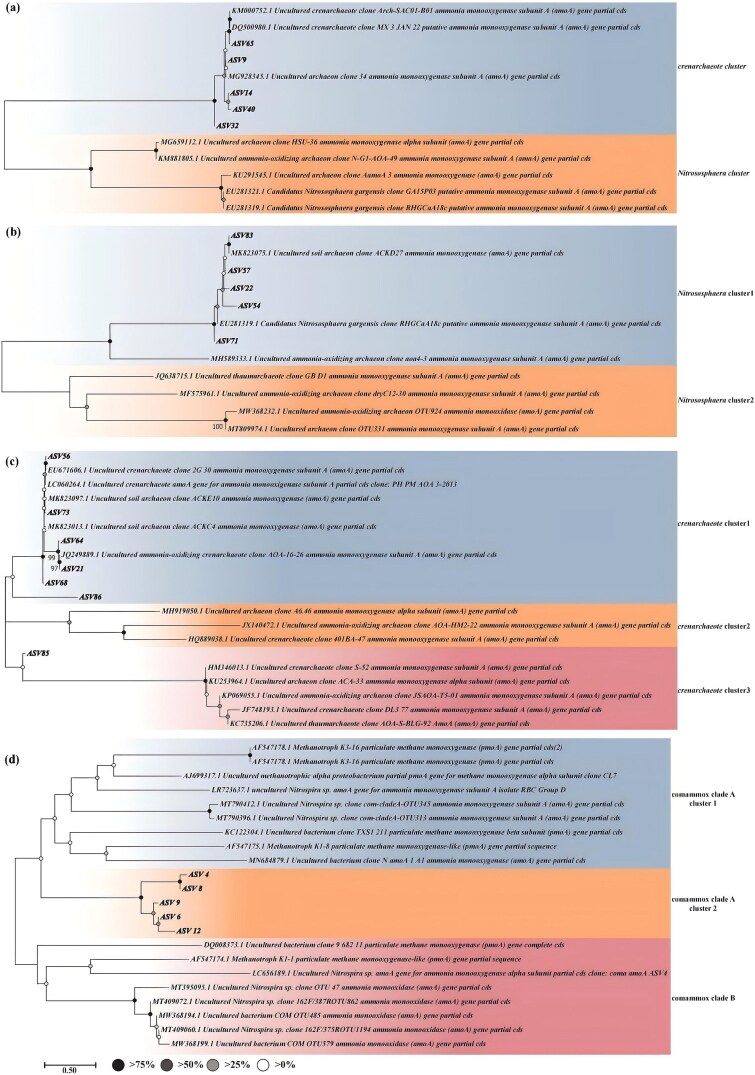
Phylogenetic trees of various AOB species labeled by ^13^C. (a) AOA groups are capable of organic and inorganic metabolism. (b) AOA groups are only capable of inorganic metabolism. (c) AOA groups are only capable of organic metabolism, (d) comammox.

We enumerated the ^13^C-DNA-enriched fractions and performed functional gene sequencing of AOA and comammox based on the ^13^C-DNA content in soils without inhibitor or Ace treatment. We clustered the sequences into ASV and selected the significantly differentially expressed ASV (100% clustering) based on the ^13^C-DNA and ^12^C-DNA abundances (Fig. 5). Significantly upregulated ASVs in soils without inhibitor and Ace treatments suggest the capacity for both organic and inorganic metabolism. ASVs upregulated only in the absence of inhibitor indicate a potential for inorganic metabolism, whereas those significantly enriched exclusively in soils without Ace imply an ability to utilize organic substrates. The phylogenetic trees of the ^13^C-labeled species showed that the AOA capable of inorganic metabolism alone belonged to *Nitrososphaera* spp., while the AOA capable of both inorganic and organic metabolism had a similar developmental distance from *Nitrososphaera* spp. but belonged to phylum *Crenarchaeota* (Fig. 5a and b). In contrast, most of the AOA that belonged to the phylum *Crenarchaeota* could only perform organic metabolism (Fig. 5c). We grouped comammox ASVs that exhibited significantly increased abundance under different treatments into a unified phylogenetic framework. Phylogenetic analysis revealed that these ASVs clustered within clade A of comammox. However, they diverged near the basal position of the clade, clearly separating from previously characterized members of clade A (Fig. 5d).

### Relative contributions of ammonia-oxidizing archaea, ammonia-oxidizing bacteria, and complete ammonia oxidizers to upland soil nitrification

**Figure 6:**
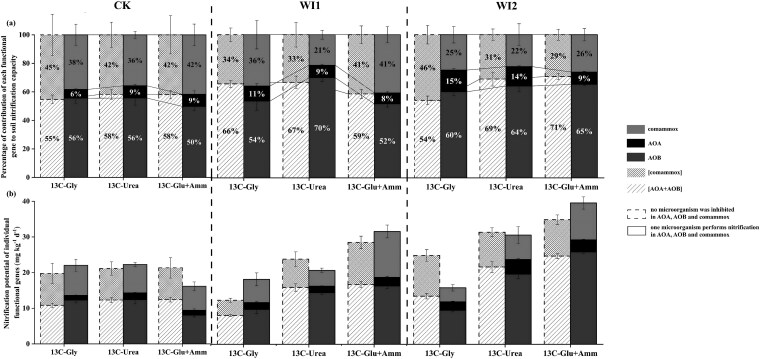
Contributions of AOA, AOB, and comammox to soil nitrification. (a) Percentage contributions to soil nitrification. (b) Soil nitrification potential under various inhibitor treatments. Sim: simvastatin; Ace: acetylene; Gly: glycine; Glu+Amm: glucose plus ammonium chloride. Error bars indicate standard deviation, *n* = 3.

We applied isotope tracer incubation using the multiple inhibitor method for 28 days to estimate the relative contributions of AOA, AOB, and comammox to the soil nitrification potential. Figure 6 shows that under conditions where AOA, AOB, and comammox were not inhibited, the relative contribution of [AOA + AOB] to nitrification in CK soil ranged from 55% to 58%, corresponding to a nitrification potential of 10.8–12.4 mg kg^−1^ d^−1^. In comparison, [comammox] accounted for 42%–45%, with a potential of 8.8–8.9 mg kg^−1^ d^−1^, indicating that these two functional groups contributed at comparable levels in the control soil. However, when non-target bacteria were suppressed, the contribution pattern was markedly reorganized: the contribution of AOA decreased to 6%–9% (1.3–1.9 mg kg^−1^ d^−1^), AOB increased to 50%–56% (8.1–12.4 mg kg^−1^ d^−1^), while comammox remained at 38%–42% (6.7–8.4 mg kg^−1^ d^−1^). The changes in contribution rates were consistent with those observed for nitrification potential (Fig. 6), indicating that shifts in functional partitioning were directly reflected in overall nitrification capacity.

Across soil types, long-term fertilization substantially altered the functional distribution pattern. In WI1 and WI2 soils, the contribution of [AOA + AOB] increased to 54%–71%, with nitrification potentials of 7.3–22.4 mg kg^−1^ d^−1^, exceeding those in CK. In contrast, the contribution of [comammox] declined to 29%–46%, with potentials of 3.77–10.4 mg kg^−1^ d^−1^, lower than in CK. Further analysis revealed that AOB became the dominant contributor in WI1 and WI2 (52%–70%, 8.6–23.4 mg kg^−1^ d^−1^). Moreover, total nitrification potential in CK soil was relatively insensitive to the addition of different exogenous substrates, whereas in WI1 and WI2 soils, the addition of ^13^C-Gly, ^13^C-Urea, and ^13^C-Glu + Amm enhanced total nitrification potential, suggesting that long-term fertilization strengthened the soil’s functional responsiveness to external carbon and nitrogen inputs.

**Figure 7:**
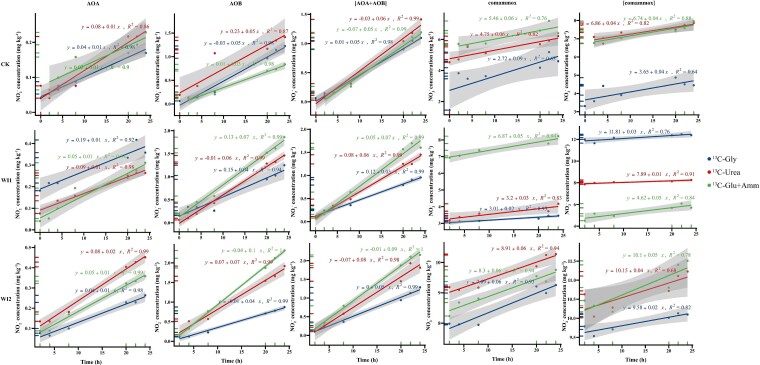
Linear regression of NO_2_^−^ or NO_3_^−^ in samples tack at 2, 4, 8, 20, 22, and 24 h based on sampling time. Sim: simvastatin; Ace: acetylene; Gly: glycine; Glu+Amm: glucose plus ammonium chloride.

Temporal changes of NO_2_^−^ and NO_3_^−^ concentrations varied markedly under different ^13^C-labeled carbon substrate amendments (Fig. 7). In most treatments (CK, WI1, and WI2), NO_2_^−^ concentrations (representing the activity of AOA or AOB) increased linearly over time, with R^2^ values generally exceeding 0.9. Among the tested substrates, the highest nitrite accumulation was observed with ^13^C-Gly and ^13^C-Glu + Amm treatments, indicating enhanced ammonia oxidation activity. The combined contribution [AOA + AOB] displayed similar linear trends, with significantly greater NO_2_^−^ production under ^13^C-Gly and ^13^C-Glu + Amm compared to ^13^C-Urea, particularly in WI2 soils. Nitrate production, representing comammox activity, exhibited distinct carbon source-dependent patterns. In the ^13^C-Glu + Amm treatment, NO_3_^−^ concentrations increased rapidly over time in both [comammox] (AOA and AOB active) and comammox (AOA and AOB inhibited) pathways, with the steepest slopes observed across all treatments. In contrast, lower NO_3_^−^ accumulation attributable to comammox was observed under the ^13^C-Gly and ^13^C-Urea treatments, with little or no increase detected in certain samples (such as WI1).

## Discussion

We emphasize that the interpretation of treatment effects was primarily based on relative differences among treatments rather than absolute values. Potential non-specific or incomplete inhibition may influence the observed ^13^C labeling patterns and the inferred metabolic interactions. In interpreting the results, we carefully considered the potential non-specific or indirect effects of the inhibitors; therefore, they were treated as operational tools rather than strictly pathway-specific probes. In this study, the definition of the “heavy fraction” was primarily based on its density position and the relative enrichment pattern of functional genes in high-density fractions, rather than on quantitative analysis of the overall DNA peak. It should also be noted that the findings of this study were mainly derived from controlled laboratory conditions, including constant temperature, stable moisture content, and relatively sufficient and homogeneous substrate supply. However, under actual field conditions, soil moisture, temperature, and substrate inputs often exhibit pronounced spatial and temporal heterogeneity. Consequently, the results presented here primarily reflect relative response patterns under controlled conditions, and their expression at the field scale may differ, meaning they cannot fully represent processes occurring in natural ecosystems.

### Contributions of ammonia-oxidizing archaea, ammonia-oxidizing bacteria, and complete ammonia oxidizers to soil nitrification under inhibitor treatments

The dynamic changes in NH_4_^+^ and NO_3_^−^ concentrations under different nitrogen sources and inhibitor treatments revealed distinct responses of soil nitrifying communities to exogenous nitrogen inputs across the three soil types (Fig. 2). Ace, a widely used inhibitor of autotrophic nitrification [23], effectively suppressed nitrification in this study, as evidenced by the inhibition of NO_3_^−^ production and the concomitant accumulation of NH_4_^+^ derived from glycine or urea mineralization. In contrast, treatments without Ace showed a sustained increase in NO_3_^−^, reflecting ongoing nitrification. Notably, Ace treatment resulted in a significant decline in AOB abundance in nearly all soils (Fig. 3), confirming its inhibitory effect on AOB populations [34]. The AOA inhibitor Sim exhibited divergent regulatory effects across soil types. In WI2 soil, Sim treatment enhanced NO_3_^−^ accumulation, which may be attributed to the increased abundance of AOB. By selectively inhibiting AOA, Sim potentially alleviated resource competition, thereby indirectly stimulating AOB nitrification activity [25].

### Organic metabolic potential and metabolic interactions between ammonia-oxidizing archaea and complete ammonia oxidizers

In CK soils amended solely with ^13^C-labeled organic substrates, ^13^C enrichment in AOB was consistently undetectable. However, under the selective inhibition of AOA by Sim, AOB exhibited significant ^13^C labeling in treatments with glycine and urea (Fig. 4). Moreover, as AOB rely exclusively on CO₂ as a carbon source [35], they depend on prior mineralization of organic substrates by other microbes. In the glucose treatment, due to its slower mineralization rate compared to glycine and urea (Fig. 7), and the likelihood that the resulting CO₂ is preferentially assimilated by AOA with higher affinity, ^13^C labeling in AOB remained undetectable.

Although heterotrophic nitrification plays a pivotal role in ecosystem nitrogen cycling, its underlying biochemical mechanisms remain largely unelucidated [9]. Phylogenetic analysis revealed that AOA with the capacity for organic metabolism are predominantly affiliated with the phylum *Thaumarchaeota*, situated near the basal branches of the archaeal lineage, and are largely heterotrophic (Fig. 5). This evolutionary placement suggests an inherent potential for organic carbon metabolism among certain AOA taxa. The comammox ASVs recovered in this study clustered phylogenetically within comammox clade A but were positioned at the basal branch of this clade, suggesting potential evolutionary and ecological significance. A basal placement typically represents an earlier-diverging lineage, indicating that these ASVs may be evolutionarily differentiated from previously cultivated and physiologically characterized comammox strains, potentially differing in substrate utilization, energy acquisition, or environmental responses, and thus representing putative functional variants. Although the concurrent detection of *amoA* and *nxrB* genes supports their affiliation with comammox *Nitrospira* and indicates the genetic potential for complete ammonia oxidation, the presence of these functional genes alone does not directly confirm that they perform complete nitrification *in situ*. In complex soil systems, gene expression and metabolic activity may be regulated by environmental conditions and microbial interactions. Therefore, these basal ASVs may exhibit differentiated or condition-dependent functional expression patterns. Collectively, they may represent previously uncharacterized comammox lineages and provide new insights into functional diversification and ecological adaptation within this group. However, further phylogenetic resolution and functional characterization of *nxrB* sequences, as well as genome-resolved or cultivation-based approaches, are required to fully resolve their taxonomic status and metabolic potential.

In CK soils treated with Ace, which fully inhibits autotrophic nitrification, neither AOA nor comammox exhibited appreciable ^13^C enrichment (^13^C < 10%), consistent with previous findings that CO₂ assimilation by AOA and AOB occurs only when ammonia oxidation is active [36]. In contrast, in soils subjected to winter irrigation with liquid organic fertilizer (WI1 and WI2), both AOA and comammox maintained substantial ^13^C enrichment even under Ace treatment. This pattern strongly suggests that WI soils harbor either a greater abundance or functionally distinct nitrifying taxa capable of sustaining higher carbon assimilation activity even under inhibitory conditions. Therefore, the observed discrepancies partly reflect differences at the community composition level. However, the present study did not further resolve fine-scale taxonomic shifts within AOA or comammox populations before and after ^13^C labeling. As a result, it remains difficult to conclusively determine whether the observed differences arise from entirely distinct taxa or from functional plasticity of the same taxa expressed under contrasting environmental contexts. Future studies employing high-resolution community profiling or time-resolved SIP experiments will be necessary to disentangle these alternative explanations.

The results of this study showed that both AOA and comammox were able to incorporate ^13^C derived from organic substrates, indicating that both groups exhibited metabolic activity during organic carbon utilization. Under non-inhibitory conditions, glycine and urea treatments markedly increased ^13^C labeling in AOA and comammox, further supporting their ability to metabolize organic nitrogen. Notably, in WI2 soil, significant ^13^C enrichment was observed in both groups despite the absence of detectable NH_4_^+^ accumulation under Ace and urea treatment, implying potential direct or synergistic urea metabolism. These findings are in line with earlier reports indicating that AOA can utilize urea directly via urease activity [37, 38]. Qin *et al.* [39] similarly demonstrated that AOA could sustain growth using urea as the nitrogen source. The direct utilization of amino acids and carbohydrates by AOA has been experimentally validated, and both compound classes ultimately feed into the TCA cycle. Genomic analysis by Zhalnina *et al.* [40] showed that *Nitrososphaera evergladensis* possesses a complete set of genes encoding enzymes required for the TCA cycle, indicating the potential for heterotrophic metabolism through this pathway. Moreover, Stieglmeier *et al.* [41] reported that TCA intermediates such as glyoxylate, oxaloacetate, and α-ketoglutarate stimulated the growth of *Nitrososphaera viennensis*, further confirming the centrality of the TCA cycle in AOA metabolism.

Importantly, Sim treatment not only suppressed ^13^C labeling in AOA but also significantly reduced labeling in comammox (Fig. 4). As Sim does not directly inhibit comammox [17], this phenomenon suggests that the two groups may not utilize organic carbon entirely independently, but instead may exhibit a certain degree of metabolic linkage. Several mechanisms may explain this observation. First, a potential cross-feeding mechanism may exist, whereby AOA transform complex organic compounds into simpler low-molecular-weight intermediates during substrate metabolism, which can then be further utilized by comammox, thereby enhancing its ^13^C incorporation. Second, the two groups may exhibit sequential rather than parallel metabolic pathways, meaning that comammox utilization of organic carbon depends on prior transformation by AOA rather than simultaneous and independent use of the same substrate. In addition, comammox may rely on CO₂ or other metabolites released during AOA metabolism as carbon sources or auxiliary substrates, thereby influencing its isotopic labeling level. However, the current DNA-SIP data alone are insufficient to distinguish among these mechanisms. Further verification is required through complete genome annotation, pure culture experiments, and supplementation experiments with organic metabolic intermediates.

### Functional drivers and microbial interaction mechanisms of soil nitrification

Integrated analysis of nitrification potential and ^13^C-tracing results indicated that AOB and comammox are the dominant functional groups mediating soil nitrification (Fig. 6). Although AOA exhibited relatively high abundance in CK soils, their nitrification potential was substantially lower than that of AOB and comammox, suggesting a functional preference towards organic substrate metabolism. AOB became the predominant ammonia oxidizers in WI1 and WI2 soils (Fig. 3), and this community shift was strongly associated with increased levels of ammonium, available phosphorus, and organic matter (Table 1). These changes in nutrient availability may have enhanced the competitive advantage of AOB while concurrently suppressing the proliferation of AOA [34, 42]. AOB dominated the ammonia-oxidizing communities in WI1 and WI2 soils, and previous studies have shown that AOB exhibit greater ecological competitiveness under conditions of elevated nitrogen availability [43, 44]. Accordingly, their nitrification potential was positively correlated with NH_4_^+^ concentrations, following the trend: ^13^C-Glu + Amm > ^13^C-Urea >^13^C-Gly (Fig. 6).

As shown in Fig. 6, the nitrification potential under the [AOA + AOB] condition was lower than that of AOA or AOB alone. This phenomenon may result from competitive interactions between AOA and AOB. Alternatively, metabolite-mediated interactions between different nitrifying microbial groups may also play a role, whereby intermediate metabolites or by-products produced by one group could inhibit the activity of the other. This inference aligns with previous observations under microcosm cultivation, which revealed a strong negative correlation between the growth of AOA and AOB [16]. In addition, the inhibitor-based approach used to differentiate nitrification pathways may itself exert non-specific effects on microbial activity, thereby influencing the estimation of nitrification potential to some extent. Moreover, proton release during nitrification may alter local pH conditions at the microscale [45, 46]. Given that AOA and AOB differ in their pH tolerance ranges and optimal growth conditions, such microenvironmental changes could lead to activity patterns under coexisting conditions that differ from those observed when each group is assessed separately. Finally, due to substantial differences in ammonia substrate affinity between AOA and AOB, their nitrification activities may occur sequentially rather than simultaneously, which could further affect the overall nitrification potential measured [11].

Although comammox contributed slightly less to nitrification than AOB, it consistently exhibited stable nitrification activity across all soil conditions, highlighting its role as a key participant in soil ammonia oxidation [47]. The coexistence of comammox with both AOA and AOB appeared to synergistically enhance overall nitrification efficiency, potentially through two mechanisms. First, our results demonstrated that the presence of AOA could promote the metabolic potential of comammox for organic substrates. Supporting this, Su *et al.* [48] reported a strong cooperative interaction between comammox and AOA under high ammonia loading, as revealed by microbial co-occurrence network analysis, suggesting metabolite sharing may contribute to enhanced nitrification efficiency. Second, phylogenetic studies have shown that comammox are closely related to AOB [4, 49], and may have acquired key nitrification genes via horizontal gene transfer. This evolutionary convergence has resulted in highly similar ammonia-oxidizing pathways between comammox and AOB, which may explain their functional resemblance and overlapping ecological roles in soil nitrification processes.

## Conclusions

This study combined inhibitor-based experiments, nitrification potential assays, and ^13^C-DNA-SIP tracing analysis to elucidate the functional differentiation and interaction mechanisms of AOA, AOB, and comammox under upland soils. AOB and comammox were identified as the primary functional groups driving soil nitrification. Among them, AOB exhibited a clear competitive advantage under high nitrogen conditions, whereas comammox maintained stable activity across all soils. Differences in community structure under distinct fertilization regimes further influenced their functional expression. The ^13^C tracing results further indicated that both AOA and comammox possess the capacity to utilize organic carbon, and potential metabolic coupling or cross-feeding interactions may occur between them. However, as this study was conducted under controlled conditions and the inhibitors applied may exert non-specific effects, the proposed mechanisms require further validation.

## Supplementary Material

ycag122_supplement_materials

## Data Availability

The datasets generated during and/or analysed during the current study are available in the National Center for Biotechnology Information (NCBI) repository, https://www.ncbi.nlm.nih.gov/, under accession number PRJNA819570.
