# Supplementary material for: Metabolic potential and contributions of ammonia-oxidizing microorganisms and complete ammonia oxidizers to soil nitrification in upland soils of northern China
Source: ISME Commun. 2026 May 7;6(1):ycag122. doi: 10.1093/ismeco/ycag122 (PMC13219743; doi:10.1093/ismeco/ycag122)
Supplement: ycag122_supplement_materials [file ycag122_supplement_materials.docx]

Metabolic potential and contributions of ammonia-oxidizing microorganisms and comammox to soil nitrification in upland soils of northern China

**Jiuwei Song^a,b^, Qichun Zhang^a*^, Ahmed A. A. Aioub^c^, Longda Gong^a^**

*^a^ State Key Laboratory of Soil Pollution Control and Safety, Zhejiang Provincial Key Laboratory of Agricultural Resources and Environment, Zhejiang University, Hangzhou, 310058, P.R China*

*^b^* *Jiangsu Key Laboratory for Bioresources of Saline Soils, Jiangsu Provincial Key Laboratory of Coastal Wetland Bioresources and Environmental Protection, Jiangsu Synthetic Innovation Center for Coastal Bio-agriculture, Yancheng Teachers University, Kaifang Road 50#, Yancheng 224002, P.R. China*

*^c^ Zagazig University*

**Fig. S1

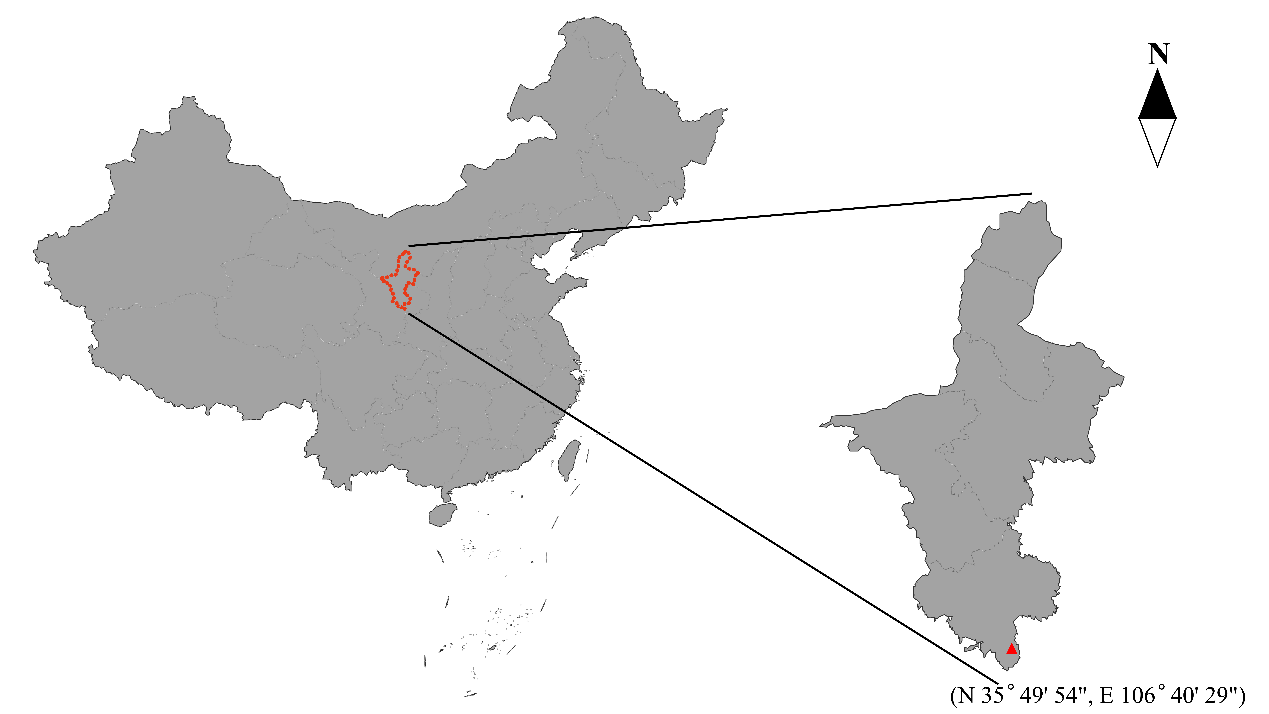
**

**Fig. S1** The map of the location where the experiment was conducted

**Table S1** Batch experiments of ^13^C and ^12^C label

| Treatments | Levels of additive |
| --- | --- |
| ^13^C-Gly | ^13^C Glycine (100 mg C kg^−1^ and 50 mg N kg^−1^) |
| ^13^C-Gly+Ace | ^13^C Glycine (100 mg C kg^−1^ and 50 mg N kg^−1^) + Acetylene (1% v/v) |
| ^13^C-Gly+Sim | ^13^C Glycine (100 mg C kg^−1^ and 50 mg N kg^−1^) + Simvastatin (12.5 mg g^−1^) |
| ^13^C-Urea | ^13^C Urea (100 mg C kg^−1^ and 200 mg N kg^−1^) |
| ^13^C-Urea+Ace | ^13^C Urea (100 mg C kg^−1^ and 200 mg N kg^−1^) + Acetylene (1% v/v) |
| ^13^C-Urea+Sim | ^13^C Urea (100 mg C kg^−1^ and 200 mg N kg^−1^) + Simvastatin (12.5 mg g^−1^) |
| ^13^C-Glu+Amm | ^13^C Glucose (100 mg C kg^−1^) + NH_4_Cl (50 mg N kg^−1^) |
| ^13^C-Glu+Amm+Ace | ^13^C Glucose (100 mg C kg^−1^) + NH_4_Cl (50 mg N kg^−1^) + Acetylene (1% v/v) |
| ^13^C-Glu+Amm+Sim | ^13^C Glucose (100 mg C kg^−1^) + NH_4_Cl (50 mg N kg^−1^) + Simvastatin (12.5 mg g^−1^) |
| ^12^C-Gly | ^12^C Glycine (100 mg C kg^−1^ and 50 mg N kg^−1^) |
| ^12^C-Gly+Ace | ^12^C Glycine (100 mg C kg^−1^ and 50 mg N kg^−1^) + Acetylene (1% v/v) |
| ^12^C-Gly+Sim | ^12^C Glycine (100 mg C kg^−1^ and 50 mg N kg^−1^) + Simvastatin (12.5 mg g^−1^) |
| ^12^C-Urea | ^12^C Urea (100 mg C kg^−1^ and 200 mg N kg^−1^) |
| ^12^C-Urea+Ace | ^12^C Urea (100 mg C kg^−1^ and 200 mg N kg^−1^) + Acetylene (1% v/v) |
| ^12^C-Urea+Sim | ^12^C Urea (100 mg C kg^−1^ and 200 mg N kg^−1^) + Simvastatin (12.5 mg g^−1^) |
| ^12^C-Glu+Amm | ^12^C Glucose (100 mg C kg^−1^) + NH_4_Cl (50 mg N kg^−1^) |
| ^12^C-Glu+Amm+Ace | ^12^C Glucose (100 mg C kg^−1^) + NH_4_Cl (50 mg N kg^−1^) + Acetylene (1% v/v) |
| ^12^C-Glu+Amm+Sim | ^12^C Glucose (100 mg C kg^−1^) + NH_4_Cl (50 mg N kg^−1^) + Simvastatin (12.5 mg g^−1^) |

**Table S2** Percentage of ^13^C labeled for AOA, AOB, and comammox

| Soil | Treatments | AOA (%) | AOB (%) | comammox (%) |
| --- | --- | --- | --- | --- |
| CK | ^13^C-Gly | 14.4 | 0 | 20.1 |
|  | ^13^C-Gly+Sim | 0 | 54.1 | 12.5 |
|  | ^13^C-Gly+Ace | 0 | 0 | 8.8 |
|  | ^13^C-Urea | 41.1 | 0 | 88 |
|  | ^13^C-Urea+Sim | 0 | 75.5 | 0 |
|  | ^13^C-Urea+Ace | 0 | 0 | 7.2 |
|  | ^13^C-Glu+Amm | 70.6 | 0 | 0 |
|  | ^13^C-Glu+Amm+Sim | 27.9 | 0 | 0 |
|  | ^13^C-Glu+Amm +Ace | 0 | 0 | 0 |
| WI1 | ^13^C-Gly | 75.5 | 11.9 | 42.1 |
|  | ^13^C-Gly+Sim | 44.9 | 13 | 29.7 |
|  | ^13^C-Gly+Ace | 9.34 | 0 | 0 |
|  | ^13^C-Urea | 55.6 | 65.4 | 5.26 |
|  | ^13^C-Urea+Sim | 0 | 84.2 | 0 |
|  | ^13^C-Urea+Ace | 0 | 0 | 8.54 |
|  | ^13^C-Glu+Amm | 48.9 | 0 | 7.8 |
|  | ^13^C-Glu+Amm+Sim | 0 | 0 | 0 |
|  | ^13^C-Glu+Amm +Ace | 38.1 | 0 | 6.2 |
| WI2 | ^13^C-Gly | 10.6 | 0 | 28.9 |
|  | ^13^C-Gly+Sim | 0 | 3.1 | 0 |
|  | ^13^C-Gly+Ace | 20.5 | 0 | 32.3 |
|  | ^13^C-Urea | 0 | 61.4 | 0 |
|  | ^13^C-Urea+Sim | 0 | 86.9 | 0 |
|  | ^13^C-Urea+Ace | 80.3 | 0 | 64.2 |
|  | ^13^C-Glu+Amm | 54.2 | 0 | 50.1 |
|  | ^13^C-Glu+Amm+Sim | 0 | 0 | 0 |
|  | ^13^C-Glu+Amm +Ace | 0 | 0 | 0 |

**Table S3** Dosages of different inhibitors applied in the soil nitrification potential assay.

| Soil | Treatments | Inhibitor dosages |
| --- | --- | --- |
| ^CK^ | ^13^C-Gly+NaClO₃ | ^13^C Glycine+NaClO₃(10 mM) |
|  | ^13^C-Gly+NaClO₃+Sim | ^13^C Glycine+NaClO₃(10 mM)+Sim(12.5 g L^−1^) |
|  | ^13^C-Gly+NaClO₃+DMPP | ^13^C Glycine+NaClO₃(10 mM)+ (w/v) DMPP(1.5% w/v) |
|  | ^13^C-Gly+NaClO₃+Sim+DMPP | ^13^C Glycine+NaClO₃(10 mM)+Sim(12.5 g L^−1^)+ (w/v) DMPP(1.5% w/v) |
|  | ^13^C-Urea+NaClO₃ | ^13^C Urea+NaClO₃(10 mM) |
|  | ^13^C-Urea+NaClO₃+Sim | ^13^C Urea+NaClO₃(10 mM)+Sim(12.5 g L^−1^) |
|  | ^13^C-Urea+NaClO₃+DMPP | ^13^C Urea+NaClO₃(10 mM)+ (w/v) DMPP(1.5% w/v) |
|  | ^13^C-Urea+NaClO₃+Sim+DMPP | ^13^C Urea+NaClO₃(10 mM)+Sim(12.5 g L^−1^)+ (w/v) DMPP(1.5% w/v) |
|  | ^13^C-Glu+NaClO₃ | ^13^C Glucose+NaClO₃(10 mM) |
|  | ^13^C-Glu+NaClO₃+Sim | ^13^C Glucose+NaClO₃(10 mM)+Sim(12.5 g L^−1^) |
|  | ^13^C-Glu+NaClO₃+DMPP | ^13^C Glucose+NaClO₃(10 mM)+ (w/v) DMPP(1.5% w/v) |
|  | ^13^C-Glu+NaClO₃+Sim+DMPP | ^13^C Glucose+NaClO₃(10 mM)+Sim(12.5 g L^−1^)+ (w/v) DMPP(1.5% w/v) |
| ^WI1^ | ^13^C-Gly+NaClO₃ | ^13^C Glycine+NaClO₃(10 mM) |
|  | ^13^C-Gly+NaClO₃+Sim | ^13^C Glycine+NaClO₃(10 mM)+Sim(12.5 g L^−1^) |
|  | ^13^C-Gly+NaClO₃+DMPP | ^13^C Glycine+NaClO₃(10 mM)+ (w/v) DMPP(1.5% w/v) |
|  | ^13^C-Gly+NaClO₃+Sim+DMPP | ^13^C Glycine+NaClO₃(10 mM)+Sim(12.5 g L^−1^)+ (w/v) DMPP(1.5% w/v) |
|  | ^13^C-Urea+NaClO₃ | ^13^C Urea+NaClO₃(10 mM) |
|  | ^13^C-Urea+NaClO₃+Sim | ^13^C Urea+NaClO₃(10 mM)+Sim(12.5 g L^−1^) |
|  | ^13^C-Urea+NaClO₃+DMPP | ^13^C Urea+NaClO₃(10 mM)+ (w/v) DMPP(1.5% w/v) |
|  | ^13^C-Urea+NaClO₃+Sim+DMPP | ^13^C Urea+NaClO₃(10 mM)+Sim(12.5 g L^−1^)+ (w/v) DMPP(1.5% w/v) |
|  | ^13^C-Glu+NaClO₃ | ^13^C Glucose+NaClO₃(10 mM) |
|  | ^13^C-Glu+NaClO₃+Sim | ^13^C Glucose+NaClO₃(10 mM)+Sim(12.5 g L^−1^) |
|  | ^13^C-Glu+NaClO₃+DMPP | ^13^C Glucose+NaClO₃(10 mM)+ (w/v) DMPP(1.5% w/v) |
|  | ^13^C-Glu+NaClO₃+Sim+DMPP | ^13^C Glucose+NaClO₃(10 mM)+Sim(12.5 g L^−1^)+ (w/v) DMPP(1.5% w/v) |
| ^WI2^ | ^13^C-Gly+NaClO₃ | ^13^C Glycine+NaClO₃(10 mM) |
|  | ^13^C-Gly+NaClO₃+Sim | ^13^C Glycine+NaClO₃(10 mM)+Sim(12.5 g L^−1^) |
|  | ^13^C-Gly+NaClO₃+DMPP | ^13^C Glycine+NaClO₃(10 mM)+ (w/v) DMPP(1.5% w/v) |
|  | ^13^C-Gly+NaClO₃+Sim+DMPP | ^13^C Glycine+NaClO₃(10 mM)+Sim(12.5 g L^−1^)+ (w/v) DMPP(1.5% w/v) |
|  | ^13^C-Urea+NaClO₃ | ^13^C Urea+NaClO₃(10 mM) |
|  | ^13^C-Urea+NaClO₃+Sim | ^13^C Urea+NaClO₃(10 mM)+Sim(12.5 g L^−1^) |
|  | ^13^C-Urea+NaClO₃+DMPP | ^13^C Urea+NaClO₃(10 mM)+ (w/v) DMPP(1.5% w/v) |
|  | ^13^C-Urea+NaClO₃+Sim+DMPP | ^13^C Urea+NaClO₃(10 mM)+Sim(12.5 g L^−1^)+ (w/v) DMPP(1.5% w/v) |
|  | ^13^C-Glu+NaClO₃ | ^13^C Glucose+NaClO₃(10 mM) |
|  | ^13^C-Glu+NaClO₃+Sim | ^13^C Glucose+NaClO₃(10 mM)+Sim(12.5 g L^−1^) |
|  | ^13^C-Glu+NaClO₃+DMPP | ^13^C Glucose+NaClO₃(10 mM)+ (w/v) DMPP(1.5% w/v) |
|  | ^13^C-Glu+NaClO₃+Sim+DMPP | ^13^C Glucose+NaClO₃(10 mM)+Sim(12.5 g L^−1^)+ (w/v) DMPP(1.5% w/v) |
